# Nogo-Receptor 1 Deficiency Has No Influence on Immune Cell Repertoire or Function during Experimental Autoimmune Encephalomyelitis

**DOI:** 10.1371/journal.pone.0082101

**Published:** 2013-12-05

**Authors:** Sara A. Litwak, Natalie L. Payne, Naomi Campanale, Ezgi Ozturk, Jae Young Lee, Steven Petratos, Christopher Siatskas, Maha Bakhuraysah, Claude C. A. Bernard

**Affiliations:** 1 Multiple Sclerosis Research Group, Australian Regenerative Medicine Institute, Monash University, Clayton, Victoria, Australia; 2 Department of Anatomy and Developmental Biology, Monash University, Clayton, Victoria, Australia; 3 Central Clinical School, Monash University, Prahran, Victoria, Australia; Klinikum rechts der Isar der Technischen Universitaet Muenchen, Germany

## Abstract

The potential role of Nogo-66 Receptor 1 (NgR1) on immune cell phenotypes and their activation during neuroinflammatory diseases such as multiple sclerosis (MS) and its animal model, experimental autoimmune encephalomyelitis (EAE), is unclear. To further understand the function of this receptor on haematopoietically-derived cells, phenotypic and functional analyses were performed using NgR1-deficient (*ngr1-/-*) animals. Flow cytometry-based phenotypic analyses performed on blood, spleen, thymus, lymph nodes, bone marrow and central nervous-system (CNS)-infiltrating blood cells revealed no immunological defects in naïve *ngr1-/-* animals versus wild-type littermate (WTLM) controls. EAE was induced by either recombinant myelin oligodendrocyte glycoprotein (rMOG), a model in which B cells are considered to contribute pathogenically, or by MOG_35–55_ peptide, a B cell-independent model. We have demonstrated that in *ngr1-/-* mice injected with MOG_35–55_, a significant reduction in the severity of EAE correlated with reduced axonal damage present in the spinal cord when compared to their WTLM controls. However, despite a reduction in axonal damage observed in the CNS of *ngr1-/-* mice at the chronic stage of disease, no clinical differences could be attributed to a specific genotype when rMOG was used as the encephalitogen. Following MOG_35–55_-induction of EAE, we could not derive any major changes to the immune cell populations analyzed between *ngr1-/-* and WTLM mice. Collectively, these data demonstrate that NgR1 has little if any effects on the repertoire of immune cells, their activation and trafficking to the CNS.

## Introduction

Multiple Sclerosis (MS) is a chronic inflammatory disease of the central nervous system (CNS) characterized by inflammation, sharply demarcated areas of demyelination and axonal loss/damage resulting in a multiplicity of neurological deficits [1], [2]. The etiology of MS is as yet unknown but it is generally accepted that the disease is the result of an autoimmune response against CNS antigens in genetically susceptible individuals [3]–[5]. Immunological, immunohistochemical and molecular analyses of MS tissue suggest that the development of this disease is driven by a Th1+Th17-type inflammatory response, in concert with an autoantibody reaction directed against defined CNS myelin and possibly neuronal components [6]. To date, MS has been regarded as a primary demyelinating disorder and much effort has been devoted to investigate the relationship between the evolution of the lesions and clinical progression in terms of myelin destruction and repair. It has now become apparent that axonal damage is an early event during the development of lesion formation in both MS and experimental autoimmune encephalomyelitis (EAE) and is the main arbiter of permanent clinical disability [7], [8].

Unlike the peripheral nervous system, regenerative nerve fiber growth and structural plasticity are limited in the adult CNS following insult [9], [10]. Notably, the limited ability of the axon to regenerate within the CNS has been attributed to the presence of myelin-associated inhibitory factors (MAIFs), present as extracellular debris components of degenerative myelin [11], [12]. In addition to astroglial scars containing chondroitin sulphate proteoglycans, the presence of MAIFs, such as Nogo-A, oligodendrocytes-myelin glycoprotein (OMgp) and myelin-associated glycoprotein (MAG) contribute to an environment impenetrable to axonal regrowth [11]. All three MAIFs are able to bind and signal through a common Nogo receptor1 (NgR1), originally described as being expressed at the neuronal membrane. NgR1 is a glycosylphosphatidylinositol (GPI)-anchored protein that complexes with TROY or p75^NTR^ and LINGO-1 co-receptors, triggering an intracellular cascade that leads to cell cytoskeleton rearrangements, ultimately culminating in neurite retraction [9], [13], [14]. The emergence of Nogo-A as one of the major MAIFs [15] and the identification of Nogo-66-induced growth cone collapse via NgR1, has led to the development of strategies aimed at overcoming Nogo-A-mediated neurite growth inhibition [16]-[20], thus providing some prospect for CNS regeneration and repair for neurodegenerative diseases with profound inflammation such as MS and spinal cord injury.

We have previously reported that *nogo-a* deficient mice, animals vaccinated with Nogo 623-640 peptide, or in EAE-induced mice treated with a neutralizing anti-Nogo antibody, all displayed reduced clinical signs and histological lesions following immunization with myelin oligodendrocyte glycoprotein peptide (MOG_35–55_). Suppression of disease was associated with a switch from a pathogenic Th1 response to a protective Th2 response [16], [17]. Moreover, in chronic-active MS demyelinating lesions, the levels of both Nogo-A and NgR1 increased in surviving oligodendrocytes, reactive astrocytes and macrophages/microglia, respectively [21], [22]. This is also emulated in the spinal cord during the course of EAE [23]. Besides being present on neural cells, NgR1, along with its signaling co-receptors are expressed on macrophages and peripheral blood immune cells in both MS patients and healthy controls [24]. The finding that upon stimulation with Nogo-A, immune cells displayed reduced adhesion and enhanced migration on myelin substrates, suggests that NgR1 may also influence activation and migration of immune cells in demyelinating diseases such as EAE and MS.

In order to further assess the relevance of NgR1 in the development of encephalitogenic and pathogenic immune responses, we undertook a comprehensive phenotypic characterization of immune cells present in primary and secondary lymphoid organs of NgR1 deficient (*ngr1-/-)* mice before and after antigenic stimulation. Moreover, and in view of the purported role that NgR1 has on peripheral T, B and other antigen presenting cells (APC) [24]-[26], we have compared the susceptibility of *ngr1-/-* mice to develop EAE provoked by either MOG_35–55_ or recombinant mouse MOG (rMOG), models considered to be B cell-independent and B cell-dependent, respectively [27], [28]. We report here that the lack of NgR1 has no significant impact on the phenotype of peripheral immune cells, both before or after antigenic stimulation, or on cell trafficking to the CNS.

## Materials and Methods

### Ethics statement

All experiments involving animal use were carried out in accordance with institutional animal ethics guidelines of the National Health and Medical Research Council of Australia and specifically approved by the Monash University School of Biomedical Sciences Animal Ethics Committee (approval number SOBSA/MIS/2007/39).

### Mice

*Ngr1-/-* mice on a C57Bl/6 background were kindly donated by Professor Strittmatter and generated as described [29]. Female mice were used throughout this study at 8-12 weeks of age. In the *ngr1-/-* animals, the exon 2 of the *ngr1* gene was replaced with the Neo^R^ cassette. Wild type littermates (WTLM) were used as controls. Animals were bred and maintained at the Monash University Central Animal Services under specific pathogen-free conditions and obtained food and water *ad libitum*. For routine genotype assessment, tail genomic DNA was extracted using Puregene® DNA Purification reagents (Qiagen, Venlo, Netherlands) and PCR performed with specific primers as described in Kim et al. [29].

### Isolation of mononuclear cells

Single cell suspensions of inguinal and axillary lymph nodes, thymus and spleen were prepared by gentle dissociation into sterile fluorescence-activated cell sorting (FACS) buffer (PBS/2% BSA/0.02% sodium azide) using a 70 µm filter. Spleen cells were incubated with sterile red cell lysis buffer (Sigma-Aldrich) for 1 minute and washed twice in FACS buffer [30]. Bone marrow (BM) cells were obtained by flushing tibiae and femurs with FACS buffer and a 27-gauge needle. CNS infiltrating mononuclear cells were isolated as previously described [31].

### Flow cytometric analysis

For flow cytometric analysis, 1×10^6^ cells were incubated with cocktails of the following primary antibodies or isotype-matched controls: FITC-anti-CD3 (145-2C11), Percp-anti-CD4 (RM4-5), PE-anti-CD8 (53-6.7), Percp-anti-B220 (RA3-6B2), FITC-anti-Gr1 (RB6-8C5), PE-anti-CD11c (HL3), APC-anti-NK1.1 (PK136), PE-anti-CD45 (30-F11), APC-anti-CD11b (M1/70) (all from BD Biosciences, Franklin Lakes, NJ USA) and APC-anti-F4/80 (BM8) (eBioscience, San Diego, CA, USA). For analysis of FoxP3, cells were stained according to the manufacturer’s protocol (eBioscience, San Diego, CA). Live cell gates were estimated using 7-AAD (eBioscience) as recommended by the manufacturer. All samples were analyzed using a FACS Canto flow cytometer (BD Biosciences). Post-acquisition analysis was performed using FACSDiva software. Cell counts for each organ were determined by gating viable cells based on cell size using a Z2 Coulter Counter. Subset cell numbers for each organ were calculated by multiplying the proportion of cells within a specified gate by the number of viable cells within that organ.

### Bone marrow colony forming assay

Femurs and tibiae bones from *ngr1-/-* or WTLM controls were aseptically removed and BM flushed with sterile PBS. Single cell suspensions were prepared and 1×10^5^ mononuclear cells were seeded in triplicate in methylcellulose medium (Stem Cell Technologies, Vancouver, Canada) suitable for the growth of granulocyte-macrophage progenitors. Colony forming units (CFU) containing granulocytes (CFU-G), macrophages (M) or a mixed type (CFU-GM) were enumerated after 8 days of culture at 37°C and 5%CO_2_ using an inverted light microscope [32].

### Induction and clinical assessment of EAE

Mice were immunized with 200 µg of MOG_35–55_ peptide (GenScript, Piscataway, NJ, USA) emulsified in Complete Freund Adjuvant (CFA, Difco) containing 4mg/ml of Mycobacterium tuberculosis (Difco) subcutaneously on the inner side of both hind flanks. Mice were given 350ng of Pertussis toxin (Sigma-Aldrich) intraperitoneally on day 0 and again 48 hr later [16]. In the second part of the study, mice were immunized with 75 µg purified rMOG, comprising the extracellular domain (amino acid residues 1-117) of the mature protein [33] emulsified in CFA and administered as above. Mice were given 200 ng of Pertussis toxin (Sigma-Aldrich) intraperitoneally on day 0 and again 48 hr later. Animals were monitored daily and clinical scores were assigned according to an arbitrary clinical scale as described [34]. Mice were humanely killed upon reaching a clinical score of 4.

### Histopathology of central nervous system

Histological evaluation was performed on paraformaldehyde-fixed, paraffin-embedded lumbar spinal cord sections (5 µm). Sections were stained with haematoxylin and eosin (H&E), luxol fast blue (LFB) and Bielshowsky silver impregnation to assess inflammation, demyelination and axonal damage respectively [16], [33]. Sections were scored blind twice for semi-quantitative analysis [35]. Representative images were taken using the 20X (oil) objective lens of an Olympus Provis Ax70 microscope and an Olympus DP70 colour digital camera. Images were processed with Adobe Photoshop (Adobe, San Jose, CA).

### Immunofluorescence analysis

Following transcardial perfusion of the mice with 4% paraformaldehyde, the optic nerves from both (18 days post-immunization, dpi) rMOG-induced EAE WTLM and *ngr1*-/- mice were removed and immersion fixed with 4% paraformaldehyde overnight at 4°C. The tissues were embedded in paraffin and cut at 10 µm. For antigen retrieval, dewaxed sections were microwaved with 0.1M citrate buffer (two 5 min) and then incubated with proteinase K (20 µg/ml, 1 hr at 37°C). The sections were then post-fixed with 4% paraformaldehyde (30 min at room temperature) and incubated with blocking buffer (10% fetal calf serum, 5% normal donkey serum, 5% normal goat serum and 0.2% Triton X-100 in phosphate-buffered saline) overnight at 4°C. Tissues were then incubated with primary antibodies (mouse anti-APP (3E9), 1∶200, Thermo Scientific; rabbit anti-NF200, 1:200, Sigma-Aldrich) for 24 hr at 4°C then followed by secondary anti-mouse (Alexa Fluor 488, Life Technologies) and anti-rabbit (Alexa Fluor 555, Life Technologies), diluted at 1∶200, incubated with 4’, 6-diamidino-2-phenylindole (Life Technologies), mounted with fluorescent mounting medium and cover-slipped. All optic nerve sections were captured with a three-channel fluorescence imaging system using a confocal Nikon C1 Upright microscope with a 60X objective lens. The captured 16-bit images were then converted to jpeg files with ImageJ and formatted using Adobe Photoshop CS5.

### Splenocyte proliferation assay and cytokine analysis

Splenocyte proliferation assays were performed at 18 and 45 dpi as previously described [33]. For cytokine analysis, 2.5×10^5^ splenocytes were stimulated with 20 µg/ml rMOG or 10 µg/ul antiCD3/antiCD28 for 48 hr. Supernatants were removed and incubated with IL-2, IL-4, IL-5, IFN-γ, TNF-α, IL-10 and IL-17 mouse cytokine capture beads (Cytometric Bead Array; BD Biosciences) for 2 hr at room temperature. Data were acquired using the FACS Canto II and analyzed using FCAP array software (Soft Flow).

### Anti-MOG antibody determination

Sera was collected from *ngr1-/-* and WTLM mice by cardiac puncture and the anti-rMOG antibody response was measured by ELISA at 18dpi as previously described [36], with sera diluted as follows: 1∶2000, 1∶4000 and 1:8000 for IgG and IgG1; 1∶1000, 1∶2000 and 1∶4000 for IgG2a; and 1∶200, 1∶400 and 1∶800 for IgM.

### Statistical analysis

All values are expressed as mean ± standard error of the mean (SEM). Statistical analysis was performed using Prism 5.04 (GraphPad Software). Unless otherwise specified, statistical comparison between genotypes was performed using a Wilcoxon-Mann-Whitney comparison test. A p value less than 0.05 was considered statistically significant.

## Results

### Phenotype of immune cells and bone marrow colony forming capacity of naïve *ngr1-/-* mice

**Figure 1:**
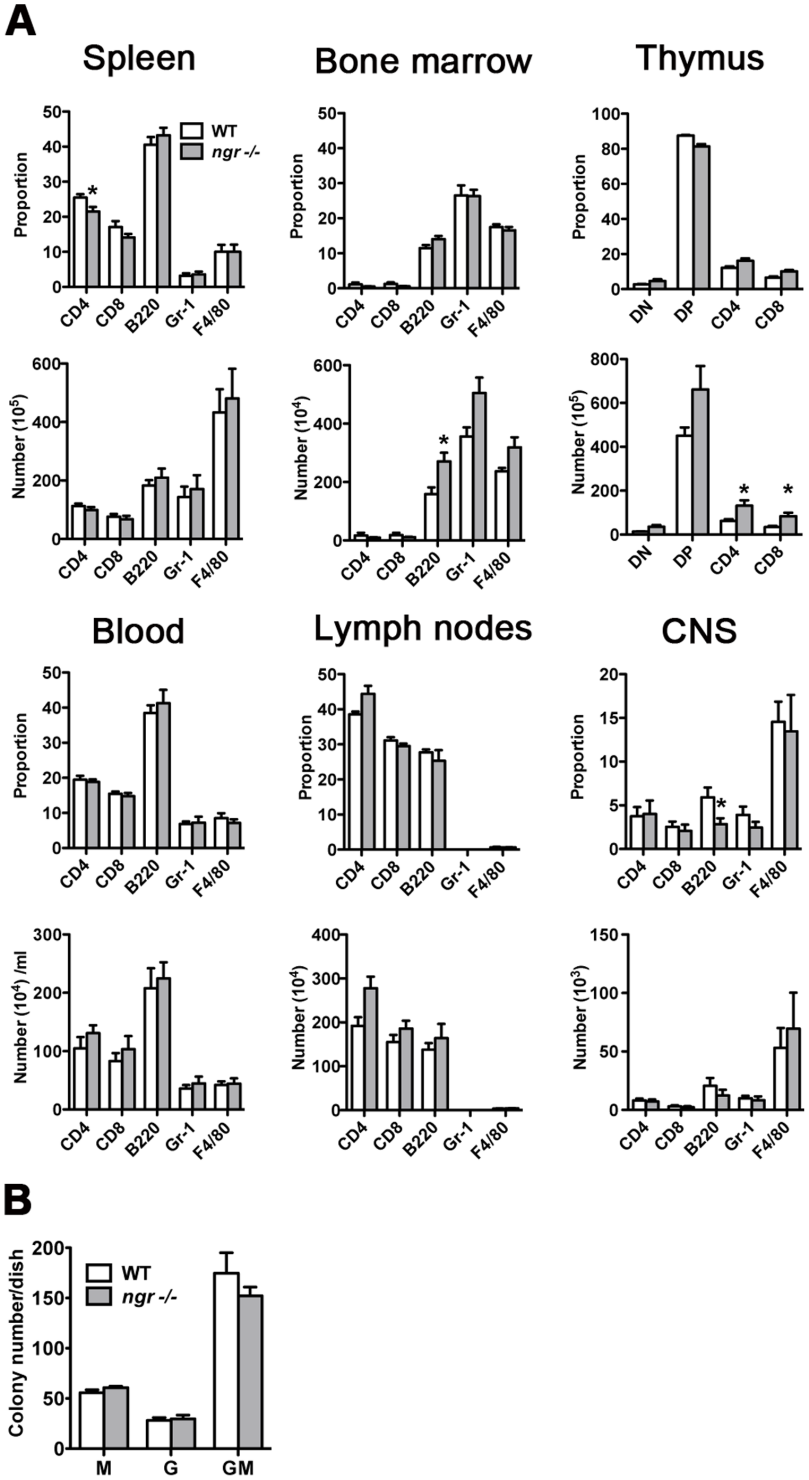
Immune-phenotype and bone marrow (BM) progenitor status of naïve *ngr1-/-* and WT mice. (**A**) Comparative flow cytometric analysis of single cell suspension from spleen, BM, thymus, blood, lymph nodes and central nervous system (CNS) associated mononuclear cells. Proportion and total number of CD3^+^CD8^+^ and CD3^+^CD4^+^ T cells, B220^+^ B cells, Gr-1^+^ granulocytes and F4/80 (Gr-1^lo^F4/80^+^) monocyte/macrophage are shown. Data represent mean ± SEM (n = 8-11). *p<0.05 Mann-Whitney test. (**B**) Quantification of BM-derived colony number in naïve *ngr1-/-* and WT mice**.** After 8 days on methylcellulose culture, BM-derived progenitors from *ngr1-/-* animals were capable of producing granulocytes (G); macrophages (M) and mixed (GM) colonies at comparable numbers to WT mice. Bars represent mean colony number/plate ± SEM (n = 3).

The presence of NgR1 and its reported role on the migration of particular leukocyte subsets [37] suggest that the receptor may contribute to the signaling cascades of these immune cells during the initial events leading to CNS autoimmunity. As a preliminary step towards understanding the impact of NgR1 on the development of EAE, we first investigated the proportion and total number of various immune cell subsets in primary and secondary lymphoid tissues as well as the CNS and blood of naïve *ngr1-/-* mice and their WTLMs. Specific markers were used to define each population, namely, CD4/CD3 and CD8/CD3 for assessing T lymphocytes, B220 for B lymphocytes, NK1.1 for natural killer cells, and Gr-1/F4/80 for granulocytes and monocytes/macrophages. Figure 1A shows the proportion and number of CD4^+^, CD8^+^, B220^+^, Gr1^+^ and F4/80^+^ cells in all six organs analyzed. Overall, the immune phenotype of *ngr1-/-* was comparable to that of the WTLM mice. However, a slight but significant decrease in the proportion of CD3^+^CD4^+^ T helper cells was observed in the spleens of *ngr1-/-* compared to those from WTLM mice (*ngr1-/-* 21.5±1.3% vs WT 25.5±1.0; p = 0.03, n = 8). While the proportions of CD3^+^CD8^+^ cytotoxic T lymphocytes and CD3^+^NK1.1^+^ NKT cells were also reduced in the spleen of *ngr1-/-* mice (14.1±0.2% and 1.4±0.1%, respectively), these values were not significantly different from that of WTLM mice (17.1±1.7% and 1.6±0.2% respectively; n = 8, Fig. 1A and data not shown).

Compared to the BM compartment of WTLM mice, *ngr1-/-* mice showed an increase in the number of B220^+^ cells (*ngr1-/-* 271.0±29.4×10^4^ vs WTLM 159.0±22.6×10^4^, p = 0.02, n = 5), Gr-1^+^ cells (*ngr1-/-* 504.5±53.2×10^4^ vs WTLM 355.9±31.2×10^4^, NS, n = 5) and F4/80^+^ (*ngr1-/-* 318.6±34.6×10^4^ vs WTLM 236.6±11.5×10^4^, NS, n = 5). There was also a significant increase in the number of single positive thymocytes of *ngr1-/-* mice, for both the CD4^+^ (*ngr1-/-* 1319.9±243.6×10^5^ vs WTLM 626.1±77.2×10^5^; p = 0.03, n = 5) and CD8^+^ (*ngr1-/-* 833.9±159.6×10^5^ vs WTLM 341.5±54.0×10^5^; p = 0.02, n = 5) subsets. No statistically significant differences in the proportion or number of immune cell subsets, was found in the blood or lymph nodes. As revealed by flow cytometric analysis, no changes were found in CD4^+^CD25^+^FoxP3^+^Tregs in the spleen of naïve *ngr1-/-* and WTLM mice with respect to the proportion (9.1±1.1% vs 9.2±0.2%) and number (109.9±11.6×10^4^ vs 96.0±25.5 ×10^4^) respectively (n = 5 mice per group, data not shown).

We next determined the proportion and number of T cells, B cells, granulocytes and monocytes/macrophages in the CNS of non-immunized *ngr1-/-* and WTLM mice. In contrast to that observed in the BM, a significantly reduced proportion of B220^+^ cells was detected in the CNS of *ngr1-/-* mice as compared to WTLM mice (*ngr1-/-* 2.9±0.7% vs WT 5.9±1.9%; p = 0.04, n = 8) (Fig. 1A), while the expression of CD4, CD8, Gr-1, F4/80, NK1.1 and CD11c (Fig. 1A and data not shown) was similar between the two groups of mice.

Given the increased number of Gr-1^+^ and B220^+^ cells in the BM of *ngr1-/-* mice together with the recent report that BAFF/BLyS, a factor important for B-cell survival (implicated in autoimmunity) can bind with high affinity to NgR1 to induce signal transduction [38], we assessed the granulocyte progenitor cell status from the BM of naïve *ngr1-/-* mice using a colony-forming assay. Eight days after plating BM cells from both *ngr1-/-* and WTLM mice, the total number of CFU-Granulocyte (G), CFU-Macrophage (M) or CFU-GM mixed colonies in each culture dish was quantified. As shown in Figure 1B, no differences in G, M and GM colony forming number were found between *ngr1-/-* and WTLMs, suggesting that myeloid progenitor development in the NgR1 deficient mice is not perturbed.

### Clinical and histological signs of MOG_35–55_-induced EAE in *ngr1-/-* mice

**Figure 2:**
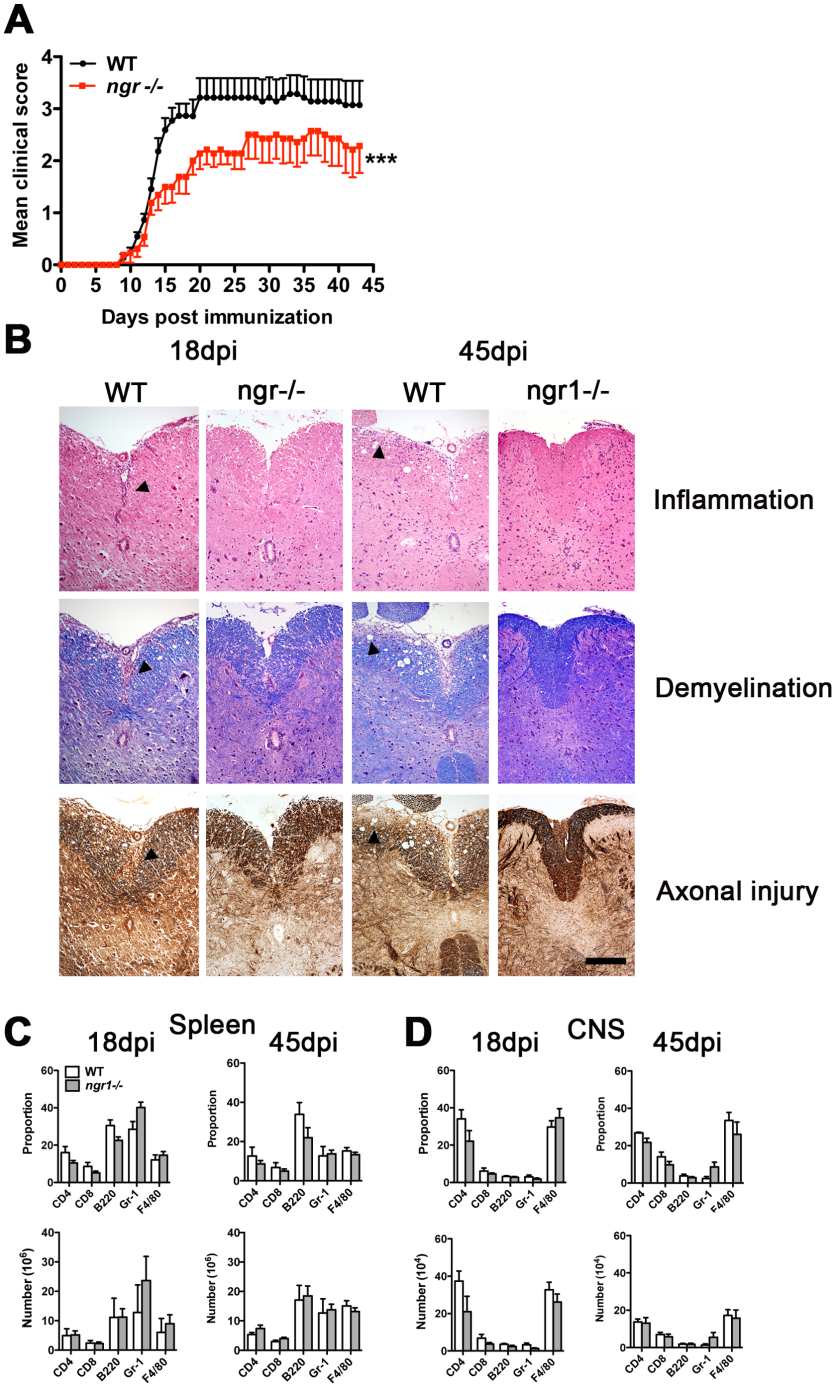
Reduced severity of MOG_35__–55_ peptide-EAE in *ngr1-/-* mice. (**A**) EAE was induced by immunization with MOG_35–55_ peptide and animals were scored daily for disease clinical manifestations. *ngr1-/-* mice presented a less severe clinical disease than WT mice. Data were pooled from 2 independent experiments (n = 11-13; mean ± SEM). ***p<0.05, two-way ANOVA. (**B**) Representative lumbar-thoracic spinal cord sections stained with hematoxylin-eosin for inflammation, luxol fast blue for demyelination and Bielschowsky silver impregnation for axonal damage. Histological examination was performed at 18 and 45 days post-immunization (dpi). Compared to WT controls, a trend towards reduced inflammation, demyelination and axonal damage could be observed in *ngr1-/-* spinal cords (magnification 20X, scale bar = 200 µm). (**C-D**) Flow cytometric analysis of spleen **(C)** and central nervous system (CNS) (**D**) mononuclear cells at 18 and 45 dpi. The proportion and number of immune cell populations analyzed did not differ significantly between *ngr-/-* and WT mice. Data represent mean ± SEM (n = 3-5).

Since no major differences in immune cell subsets were identified between naïve *ngr1-/-* and WTLM animals, we next sought to determine whether the lack of NgR1 could impact on the trafficking of leukocytes to the CNS, thereby affecting the development of EAE provoked by MOG_35–55_. To this effect, we first compared the development and severity of EAE in *ngr1-/-* and WTLM mice. There was a small but significant delay in the onset of disease in *ngr1-/-* mice compared to WTLM (*ngr1-/-* day 10.5±0.3 vs WTLM day 11.9±0.5; p = 0.02, n = 11-13) (Fig. 2A and Table 1). Disease severity was also significantly reduced in the *ngr1-/-* group, the mean EAE scores at day 18 being 1.7±0.3 and 3.1±0.4 for *ngr1-/-* and WTLM mice, respectively (p = 0.04). This was associated with a decrease in disease incidence at 18 dpi (*ngr1-/-* 23% vs WTLM 100%) and maximum clinical score (*ngr1-/-* 2.0±0.3 vs WTLM 3.1±0.4). By the chronic stage of the disease (45 dpi), the incidence of EAE was equal in both groups of mice (100%). However, the mean clinical score as well as the cumulative score of *ngr1-/-* mice were reduced, albeit not significantly, compared to WTLM (*ngr1-/-* 2.6±0.7 and 75.7±13.5 vs WTLM 3.6±0.7 and 111.6±16.3, respectively; n = 7).

**Table 1:** Clinical and histological outcome of MOG_35–55_ EAE in *ngr1^−/−^.*

	18dpi		45dpi	
	WT	*ngr1^−/−^*	WT	*ngr1^−/−^*
**Incidence**	11/11	3/13	7/7	7/7
**Death of severe disease**	3/11	0/13	3/7	2/7
**Onset day**	10.5±0.3	11.9±0.5*	10.0±0.4	12.3±0.9*
**Maximum score**	3.1±0.4	2.0±0.3	3.9±0.5	3.1±0.5
**Cumulative score**	17.5±2.0	9.0±2.1*	111.6±16.3	75.7±13.5
**Disease duration**	8.5±0.3	5.8±0.7**	34±0.4	31±1.2
**Inflammation score**	2.4±0.2	1.6±0.4*	1.7±0.2	1.3±0.2
**Demyelination score**	2.1±0.1	1.2±0.4	1.4±0.3	0.8±0.1
**Axonal injury score**	2.4±0.3	1.4±0.4	1.9±0.3	1.2±0.2*

Data represent mean ± SEM. *p<0.05, **p<0.01. Wild Type (WT) and Nogo Receptor 1 deficient (*ngr1^−/−^)* values are shown for the peak (18 days post-immunization, dpi) and the chronic (45dpi) stage of MOG peptide (MOG_35–55_ )-EAE.

Blind histopathological evaluation of H&E stained spinal cord sections from WTLM mice at 18 and 45 dpi revealed typical extensive mononuclear inflammatory cell infiltration (Fig. 2B). Luxol fast blue and Bielschowsky silver staining also showed widespread myelin loss and axonal injury, respectively. In comparison, *ngr1-/-* animals exhibited significantly decreased inflammation at 18 dpi with associated protection of the myelin architecture and significantly reduced axonal damage at 45 dpi (Fig. 2B).

To determine if the composition of immune cells differed between *ngr1-/-* and WTLM mice during the course of EAE, the proportion and total number of CD4^+^, CD8^+^, B220^+^, Gr-1^+^ and F4/80^+^ cells present in the spleen and CNS of these mice were analyzed by flow cytometry at 18 and 45 dpi. As indicated in Figure 2C and D, no significant differences in the number and proportion of T cells, B cells or granulocytes/monocytes were found between *ngr1-/-* and WTLM controls at the two time points examined. Similarly, the BM, thymus and lymph nodes of *ngr1-/-* and WTLM mice showed no detectable differences in T cells, B cells or granulocytes/monocytes (data not shown). On the basis of these findings, it would appear that under inflammatory conditions, the presence of NgR1 on immune cells has little or no influence on their migratory behavior in the CNS.

### Clinical and histological signs of rMOG_–_-induced EAE in *ngr1-/-* mice

**Figure 3:**
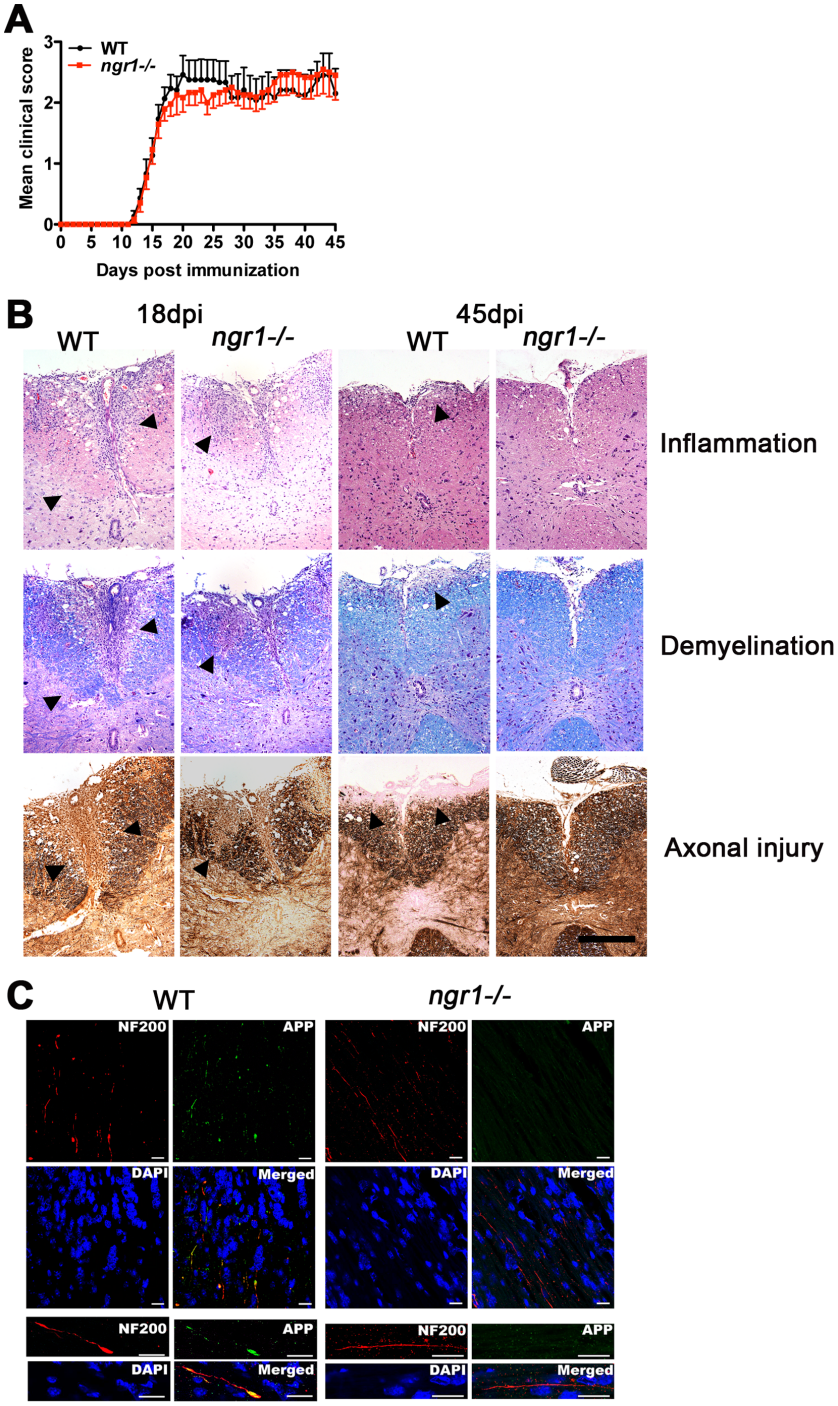
Susceptibility of *ngr1-/-* mice to develop EAE provoked by rMOG. (**A**) EAE was induced by immunization with the extracellular domain of mouse rMOG and animals were scored daily for disease clinical manifestations. There were no discernible variation in the severity of disease between *ngr1-/-* and WT mice. Data were pooled from 3 independent experiments (n = 19-22; mean ± SEM). (**B**) Representative lumbar-thoracic spinal cord sections stained with hematoxylin-eosin for inflammation, luxol fast blue for demyelination and Bielschowsky silver impregnation for axonal damage. Histological examination was performed at 18 and 45 days post-immunization (dpi). In contrast to WT controls, reduced axonal damage in *ngr1-/-* spinal cords could be observed at the chronic stage (45dpi) of EAE (magnification 20X, scale bar = 200 µm). (**C**) Amyloid precursor protein (APP)-positive, NF-200-positive axons (arrow) near DAPI-positive inflammatory infiltrates in optic nerves from rMOG-induced WT mice at 18 dpi (left, score 3). Reduced APP-immunopositive axons in optic nerves from rMOG-induced *ngr1*-/- mice (right, score 2.5). Scale bar  =  20 µm.

We next assessed whether immunization with rMOG would influence the susceptibility and development of EAE in *ngr1-/-* mice as well as their peripheral immune response. In contrast to the MOG_35–55_, the rMOG protein (consisting of the ectodomain) requires intracellular processing for its encephalitogenic activity [39], thereby potentiating a vigorous B cell-dependent response [28]. At variance with that observed when EAE was induced with MOG_35–55_, *ngr1-/-* mice developed EAE signs indistinguishable from that of WTLM animals when rMOG was used as the encephalitogen (Fig. 3A and Table 2). Consistent with this finding, histological analysis of spinal cord sections performed at 18 and 45 dpi revealed that both group of animals had extensive inflammatory lesions and associated demyelination at these two time points (Fig. 3B). Interestingly, the cohorts of *ngr1-/-* and WTLM mice examined at the chronic stage of EAE (45 dpi) were found to have reduced inflammation and demyelination as compared to 18 dpi. Notably, *ngr1-/-* mice had significantly less axonal damage than WTLM mice (*ngr1-/-* 1.5±0.2 vs WTLM 2.5±0.2; p = 0.04, n = 4) (Fig. 3B and Table 2), suggesting that NgR1 may play a role in axonal damage. As a corollary to the histological analyses performed on the mouse spinal cord tissues following rMOG-induced EAE, we further investigated the integrity of axons in the optic nerves of both *ngr1-/-* and WTLM mice at the peak stage of disease (clinical score 2.5 and 3, respectively). We found that axonal degeneration was limited in the *ngr1-/-* mice, with an absence of intense APP-positive immunostaining of dystrophic axons being present in the optic nerve (Fig. 3C). This was in contrast to substantial APP accumulation observed in degenerating axons illustrated in the optic nerves of WTLM mice at the same time point and stage of disease (Fig. 3C). These data suggest that even with the presence of extent clinical disease in the *ngr1-/-* induced through rMOG immunization, axonal preservation is still maintained within the optic nerves of these mice.

**Table 2:** Clinical and histological outcome of rMOG EAE in *ngr1^−/−^.*

	18dpi		45dpi	
	WT	*ngr1^−/−^*	WT	*ngr1^−/−^*
**Incidence**	20/20	22/24	12/12	11/12
**Death of severe disease**	0/20	0/24	1/12	1/12
**Onset day**	14.1±0.3	14.5±0.3	15.2±0.4	14.7±0.4
**Maximum score**	2.4±0.1	2.3±0.1	2.7±0.2	3.0±0.2
**Cumulative score**	8.3±0.8	8.7±1.0	67.1±9.4	66.7±7.6
**Disease duration**	4.4±0.4	4.3±0.4	29.4±2.5	27.4±2.7
**Inflammation score**	2.9±0.3	2.9±0.2	1.7±0.2	1.5±0.1
**Demyelination score**	2.4±0.2	2.3±0.2	1.3±0.2	1.1±0.1
**Axonal injury score**	2.4±0.2	2.7±0.1	2.1±0.2	1.4±0.2*

Data represent mean ± SEM. *p≤0.05. Wild Type (WT) and Nogo Receptor 1 deficient (*ngr1^−/−^*) values are shown for the peak (18 days post-immunization, dpi) and the chronic (45dpi) stage of recombinant MOG-EAE.

### Phenotype of immune cells in *ngr1-/-* deficient mice immunized with rMOG

**Figure 4:**
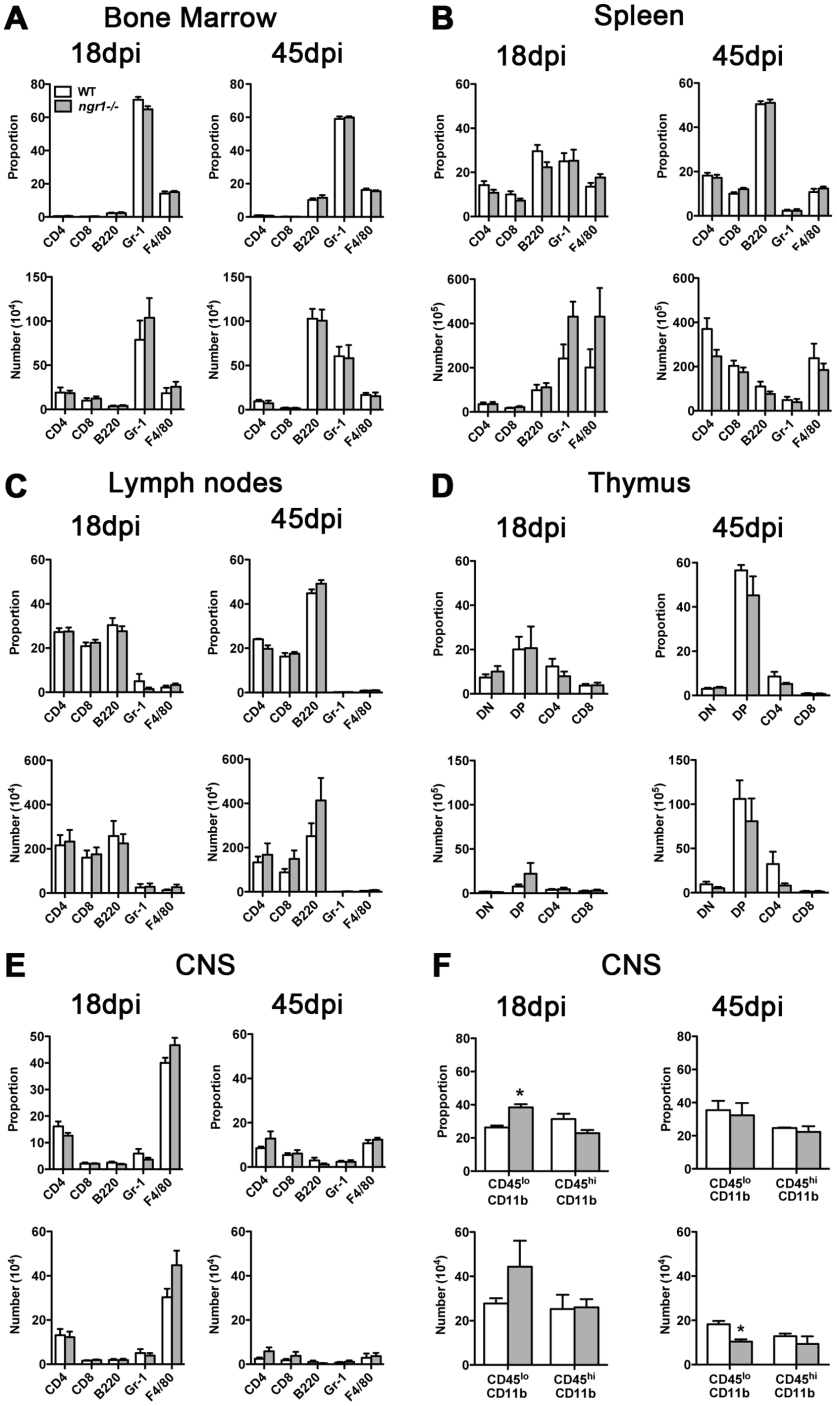
Immune-phenotype of *ngr1-/-* mice during EAE induced with rMOG. *ngr1-/-* and WT animals were immunized with rMOG and the mononuclear cells isolated at 18 (peak) and 45 (chronic) days post-immunization (dpi) were analyzed by flow cytometry. Proportion and total number of lymphocytes, granulocytes and monocyte/macrophages of bone marrow (BM; **A**); spleen, (**B**); lymph nodes, (**C**); thymus (**D**) and central nervous system (CNS; **E**) are shown. No significant differences were found between *ngr1-/-* and WT for all organs and time points examined. Data represent mean ± SEM (n = 6-11). (**F**) Further analysis of microglia (CD45^lo^CD11b^+^) and macrophages (CD45^hi^CD11b^+^) in the CNS of *ngr1-/-* and WT mice was performed. *ngr1-/-* animals presented an increased proportion of microglial cells at 18 dpi and a decreased number of macrophages at 45 dpi (n = 3-4; *p< 0.05 Mann-Whitney test).

We next examined the immune cell subsets present in various organs harvested from *ngr1-/-* and WTLM mice at 18 and 45 dpi (Fig. 4A-F). No statistical differences were found in the proportion and number of T, B, granulocytes and monocytes/macrophages between *ngr1-/-* and WTLM mice for all peripheral immunological organs examined, (Fig. 4A-E). A prevalence of Gr-1^+^ cells in the BM of both *ngr1-/-* and WTLM mice was observed at 18 dpi, the mean proportion and number of Gr-1^+^ cells for the WTLM mice being 70.6±1.8% and 150.5±14.8×10^4^ and for the *ngr1-/-* mice, 64.9±1.8% and 139.0±25.3×10^4^, respectively. At 45 dpi, these values were 59.1±1.4% and 60.4±10.8 ×10^4^ for WTLM and 59.9±0.7% and 58.2±14.9 ×10^4^ for *ngr1-/-* mice (Fig. 4A).

Interestingly and in contrast to that observed in the thymus of naïve mice, both groups of animals showed a low number of double positive thymocytes during the peak stage of EAE (*ngr1-/-* 21.9±12.1×10^5^ vs WTLM 7.6±2.3×10^5^), recovering to higher levels by 45 dpi (*ngr1-/-* 80.8±25.7×10^5^ vs WTLM 105.9±21.1×10^5^).

We then assessed the profile of resident and infiltrating immune cells present in the CNS of EAE animals. As indicated in Figure 4E, F4/80^+^ cells represented the highest proportion and number of CNS-associated immune cells at both stages of the disease, albeit in greater number at 18 dpi. Resident microglia (CD45^lo^CD11b^+^) and peripherally derived myeloid cells (CD45^hi^CD11b^+^) were also quantified, representing between 20-30% of the CD45^+^ population in the CNS of EAE mice (Fig. 4F). The proportion of microglia in *ngr1-/-* mice was increased at 18 dpi (*ngr1-/-* 34.1±1.9% vs WT 25.0±1.3%; p = 0.02) while the number was significantly reduced at day 45 in comparison with the WTLM (*ngr1-/-* 8.9±1.0×10^4^ vs WT 18.2±1.5×10^4^; p = 0.02,n = 3-4).

### Peripheral immune response to rMOG in *ngr1-/-* mice

**Figure 5:**
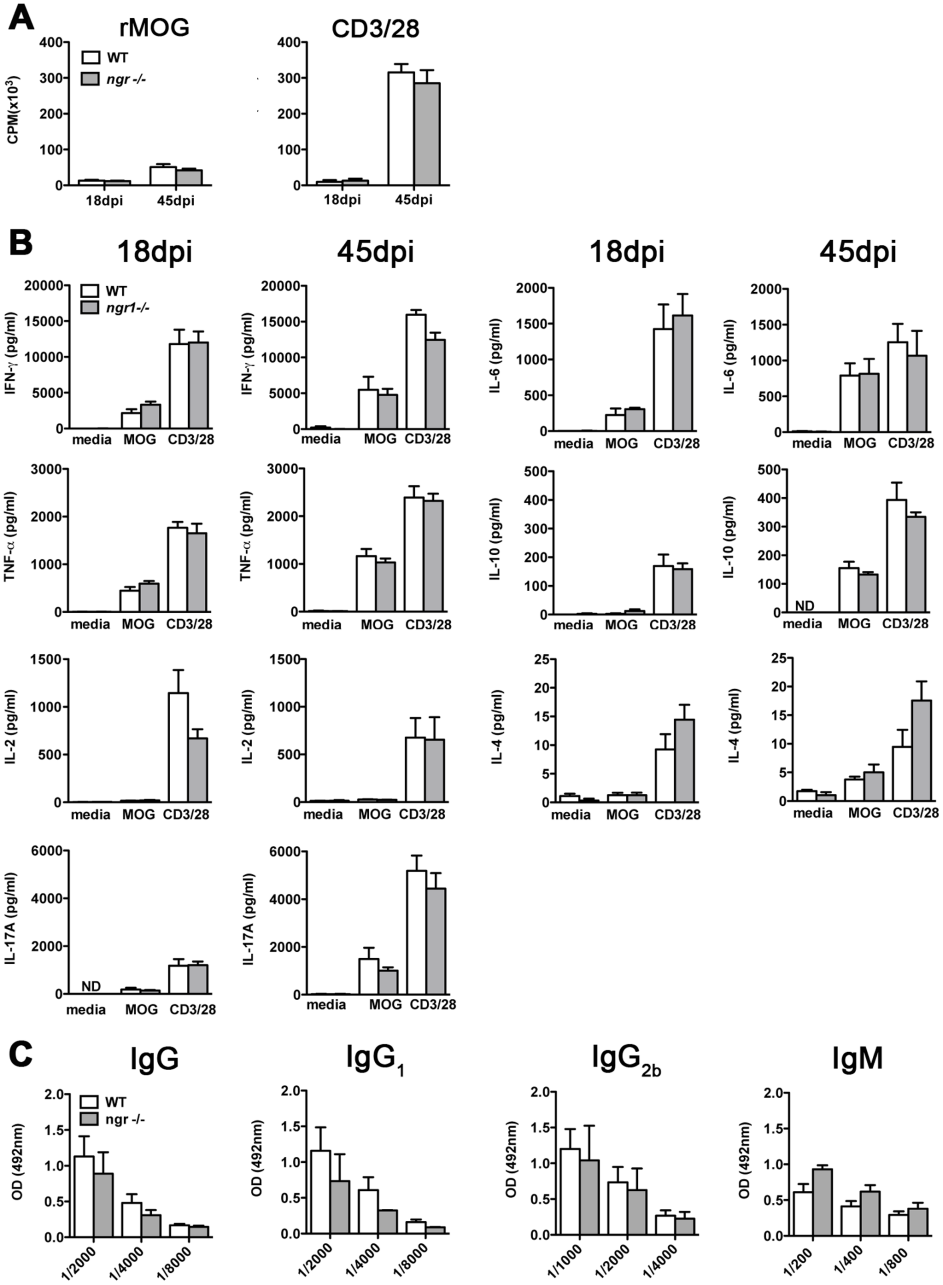
Peripheral immune response of *ngr1-/-* mice to rMOG. (**A**) *In vitro* proliferative response of *ngr1-/-* and WT splenocytes stimulated with rMOG or anti-CD3/CD28 at 18 and 45 days post immunization (dpi). *ngr1-/-* splenocytes showed an equivalent proliferative response to that of WT splenocytes. (**B**) Quantification of pro-inflammatory (INF-γ, TNFα, Interleukin (IL)-2, IL-17A and IL-6) and anti-inflammatory (IL-4 and IL-10) cytokines in supernatants derived from rMOG and anti-CD3/CD28 stimulated splenocytes cultures. *ngr1* deficiency had no impact on splenocyte cytokine production at either time points analyzed. Data represent mean ± SEM (n = 3-5). (**C**) Serum rMOG-specific IgG, IgG1, IgG2b and IgM antibody response as determined by ELISA. Data represent mean ± SEM (n = 2-6).

We have previously reported that *ngr1-/-* mice display no difference in their T cell proliferative responses or cytokine production compared to WTLM in MOG_35–55_-induced EAE [17]. Thus, to determine whether NgR1 can influence the peripheral immune response to rMOG stimulation, we first performed re-call proliferation assays to assess the *ex vivo* proliferative response of splenic T-cells to MOG and to non-specific mitogens. As shown in Figure 5, no significant difference in rMOG-specific T-cell responses was observed between the *ngr1-/-* and WTLM mice, at either time point examined. Likewise, there was no difference in T-cell proliferation when splenocytes from both groups of mice were re-stimulated with anti-CD3/CD28. Moreover, flow cytometric analysis performed 18 days post-EAE induction revealed no changes in proportion or total numbers of CD4^+^CD25^+^FoxP3^+^Tregs in the spleen and lymph nodes in the 2 cohorts of mice. The proportion of FoxP3^+^ cells in the spleen and lymph nodes of *ngr1-/-* mice was 12.6±1.2% and 11.6 ±1.8% respectively, compared to 12.4±1.9% and 10.5±1.3% for WTLM mice, respectively. The total number was 79.1±27.5×10^4^ in *ngr1-/-* spleen and 24.6±4.8×10^4^ in the WTLM lymph nodes and 48.0±9.0×10^4^ in *ngr1-/-* and 13.8±3.3×10^4^ in WTLM, respectively (n = 5 mice per group, data not shown).

Quantification of cytokines in rMOG or anti-CD3/CD28 stimulated cultures revealed that splenocyte supernatants from *ngr1-/-* and WTLM mice contained comparable levels of IFN-γ, TNF-α, IL-2, IL-17A, IL-4, IL-10 and IL-6 (Fig. 5B). Taken together, these results suggest that the deficiency in NgR1 expression has little impact (if at all) on the ability of peripheral T cells to mount both specific and non-specific immune responses.

Given that rMOG immunization has been reported to induce autoantibodies with demyelinating activity [40], [41], an assessment of the rMOG-specific IgM and IgG antibody response as well as the IgG1 and IgG2b subclasses in the sera of mice at the chronic stage of disease was performed. As reported in Figure 5C, no difference between *ngr1-/-* and WTLM mice was observed.

## Discussion

Current treatments for MS mainly target the inflammatory aspects of the disease. Novel therapeutics aimed at preventing or limiting axonal degeneration, one of the major arbiters of neurological decline in the human disease, as well as its animal model, EAE [1], [9], are therefore required. Blockade of NgR1-ligand signaling is considered one such promising approach. Nogo-A-dependent signaling has now been shown to promote the development of EAE and models of spinal cord injury [16], [29]. Together with other MAIFs and their receptors, such signaling may impede axonal growth and regeneration through a pathological lesion characterized by acute and chronic inflammatory conditions [42]. We and others have previously demonstrated that active or passive immunization designed to inhibit the biological activity of Nogo-A in autoimmune mediated demyelination, not only attenuated clinical signs, demyelination and axonal damage, but also reduced the production of pathogenic Th1-associated cytokines [16], [43]. Similarly, genetic deletion of LINGO-1, a NgR1 co-receptor, or its inactivation by treatment with an antagonizing antibody, led to functional recovery of mice with EAE. Interestingly, this effect was shown to be independent of immune-modulation [20]. Likewise, in MOG_35–55_ and MBP-induced EAE, silencing of Nogo-A with small interfering RNA limited the clinical severity of disease and promoted repair without altering the phenotype of myelin-specific T cells [18], thus bringing into question the mechanism(s) by which blockade of Nogo-A exerts a therapeutic effect.

Emerging evidence suggests that, in addition to its role in preventing axonal regrowth, NgR1-ligand signaling may also influence the behavior of immune cells. Indeed, NgR1 has been reported to be expressed on rat macrophages, human lymphocytes and monocytes as well as on a variety of inflammatory cells present in chronic active MS demyelinating lesions, implying that NgR1 may be important in modulating adherence and/or immune cell migration and function [24], [25], [37]. So far, only one report demonstrated the presence of non-neuronal NgR1 components in MS brain samples, specifically on astrocytes and microglia in demyelinating lesions [21], [22]. Expression of NgR1 components has also been identified *ex vivo* on human T cells, B cells and monocytes [24]. This, together with the demonstration that the B cell-activating factor BAFF/BLyS, a potent B cell survival factor, interacts with NgR1 [38], suggests that signaling through the MAIFs-NgR1 cascade might account for a wide range of immunological effects, although the physiological implications of these findings are yet to be fully elucidated.

In the present study we sought to determine whether *ngr1* deletion affects the repertoire and function of immune cells under naïve conditions, during the initiation of EAE and the ensuing pathophysiological responses. Given that B cells are not only able to process and present antigen [44] but also play an important functional role in MS [40], [45], two related MOG-induced MS-like disease models were used. The first, induced by MOG_35–55_ is independent of B cells, the other produced with rMOG, is B-cell dependent [28], [39], [41], [46]. As we show here, *ngr1*-deficient mice present an immune profile comparable to their WTLM counterparts under both naïve and pathological conditions, with no major differences in the proportion or total number of immune cells identified. Despite these findings, the severity of clinical signs was significantly less in *ngr1-/-* mice compared to that of WTLM mice upon immunization with MOG_35–55_, with an associated reduction in CNS tissue inflammation, demyelination and axonal degeneration. These results are in line with our very recent finding that the reduction and/or prevention of axonal degeneration in MOG_35–55_-induced EAE is associated with lower levels of the phosphorylated form of CRMP-2, a tubulin-associated protein that regulates axonal growth dynamics [17]. However, our data are in contrast to those reported by Steinback et al., who observed an increased leukocyte infiltration into the CNS of *ngr1-* and *ngr2*-deficient mice, even though neither of these receptors had any significant impact on the development and severity of EAE, nor did they influence the size and distribution of inflammatory lesions within the CNS [47]. The reason for this discrepancy is not clearly apparent but may reflect differences in the generation of the *ngr1-*deficient mice used in both studies, which have also plagued the interpretation seen in spinal cord injury paradigms [29], [48]. Alternatively, other factors such the source of the MOG peptide, the strength of the encephalitogenic challenge and/or the conditions in the animal facilities, are also variables which must be considered when interpreting all EAE studies [49].

A recent study by McDonald et al. [26] suggested that NgR1 and NgR2 expression may regulate the adherence capacity of mouse and human dendritic cells (DC) to myelin substrate, and thus play a role in the processing of myelin for antigen presentation in EAE and MS. In EAE induced by rMOG, B cells act as sophisticated and highly selective APCs that promote the differentiation of pro-inflammatory Th1 and Th17 cells [28]. Our inability to detect any clinical benefits when EAE was provoked by rMOG is therefore of particular interest, as this suggests that under the experimental conditions used, NgR1 does not affect the function of B cells. Moreover, we did not detect any difference in the T-cell proliferative responses or production of pro- and anti-inflammatory cytokines between the *ngr1-/-* and WTLM mice at either the peak or the chronic stage of EAE. Likewise, in our previous study, no changes in peripheral immune responses were observed when EAE was induced with MOG_35–55_ [17]. Taken together, these results imply that NgR1 has little (if any) influence on the APC function of B cells or the production of potentially pathogenic immune responses. While the exact mechanisms by which B-cells contribute to humoral and cellular mechanisms of MS pathogenesis are still not fully understood, B-cell targeted therapies such as Rituximab have nevertheless shown promising results in clinical trials [50]. Of particular interest to the current study, anti-CD20 treatment was recently shown to have divergent effects in EAE induced by MOG peptide versus MOG protein [28]. The clinical benefit of anti-CD20 observed only in the latter model was due to a reduction in pro-inflammatory Th1 and Th17 responses induced by activated MOG-reactive B-cells. Thus it would be of importance to investigate the susceptibility of *ngr1-/-* mice following induction of EAE with rMOG and treatment with B-cell depleting agents, and determine whether this restores the attenuation of disease that is observed in *ngr1-/-* mice immunized with MOG_35–55_.

Changes in the resident as well as infiltrating immune cells during the course of EAE are well understood. Under normal conditions or at the initial stage of EAE, resident microglial cells constitute the major immune cell population within the CNS. Once autoreactive T cells traffic to the CNS, they undergo a second round of reactivation by local APCs such as macrophages and microglia. It is this intricate interaction between cells of the immune system, which communicate via a complex network of cytokines, chemokines and adhesion molecules that shapes the nature and severity of the disease [51], [52]. During this inflammatory process, microglia/macrophages play an important role in clearance of myelin debris, eventually promoting the resolution of inflammation [53]. Recently, Fry and colleagues proposed that NgR1 might have a potential role in macrophage behavior, providing a signal for their efflux from the inflamed region upon encountering newly formed myelin patches. However, these findings were obtained by investigating the peripheral nervous system under injury conditions [25]. In line with these findings, a recent report has suggested that Nogo-66-dependent signaling mechanisms can prevent the microglial adhesion and migration response with possible implications for lesion resolution in MS [54]. In contrast to the study of Steinback et al. [47], we were unable to find differences in the myeloid and general immune cell populations at day 18 and 45 post-EAE induction, time points that correspond to the peak and to the chronic stages of the disease. Obviously, since we have only assessed the total immune cell populations present in the CNS, we cannot rule out the possibility that changes at the lesion sites may influence the trafficking of monocytes/macrophages to defined areas of the CNS tissue. In this context, it is noteworthy that during the course of MOG-induced EAE, Nogo-A and NgR are actively regulated in acute lesions, highlighting the importance of temporal and spatial changes within the inflamed CNS [23].

While both *ngr1-/-* and WTLM mice developed severe clinical signs of EAE, we observed a reduction in axonal damage and loss in the spinal cord of *ngr1-/-* mice immunized with rMOG at the later stage of the disease (45dpi) but also at the peak stage of EAE within the optic nerve, despite the presence of inflammatory demyelinating lesions. On the basis of these findings it would appear that the lack of NgR1 may influence the process of axonal preservation. However, this does not translate into a reduction in rMOG-EAE disease severity, as was demonstrated following MOG_35–55_ immunization. These results may imply that antibody-mediated destruction of the myelin sheath does not promote further axonal damage in *ngr1-/-* mice, with maintained axonal integrity allowing for the possibility of endogenous remyelination/repair to ensue. This is in line with our recent report showing that in contrast to what is observed at the peak stage of EAE, there is an equivalent proportion of demyelination in the optic nerves of both *ngr1-/-* mice and WTLM mice at earlier stages of this MS-like disease [17].

In summary, our data fail to provide any evidence for the influence of NgR1 on leukocyte subsets in the primary and secondary lymphoid organs of naïve animals. Moreover, NgR1 appears to have no obvious effect on the proportion and number of immune cell subsets present within the CNS under neuroinflammatory conditions. While we were able to observe some reduction in axonal injury in the *ngr1-/-* animals immunized with rMOG, no difference in inflammation or demyelination could be detected between such immunized mice and their WTLM mice counterparts. Further studies, especially those utilizing conditional gene targeting, may be required to dissect the role of the NgR homologues and their putative ligands on immune cell behavior during the immunopathogenesis of experimentally-induced and naturally occurring neurodegenerative diseases such as EAE and MS.
